# Supplementary figures and images for: Immune disease dialogue of chemokine-based cell communications as revealed by single-cell RNA sequencing meta-analysis
Source: Front Syst Biol. 2024 Dec 12;4:1466368. doi: 10.3389/fsysb.2024.1466368 (PMC12341984; doi:10.3389/fsysb.2024.1466368)

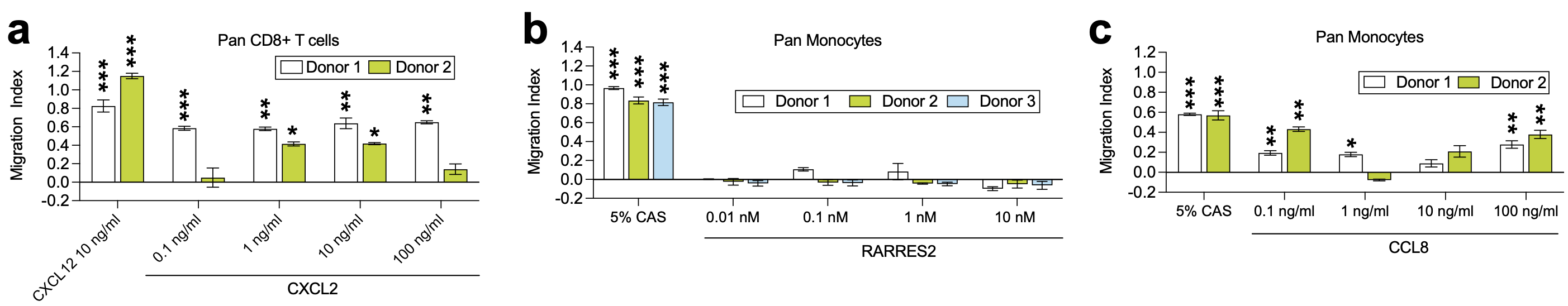

Supplement: Supplementary file 1 [file Image2.jpg]

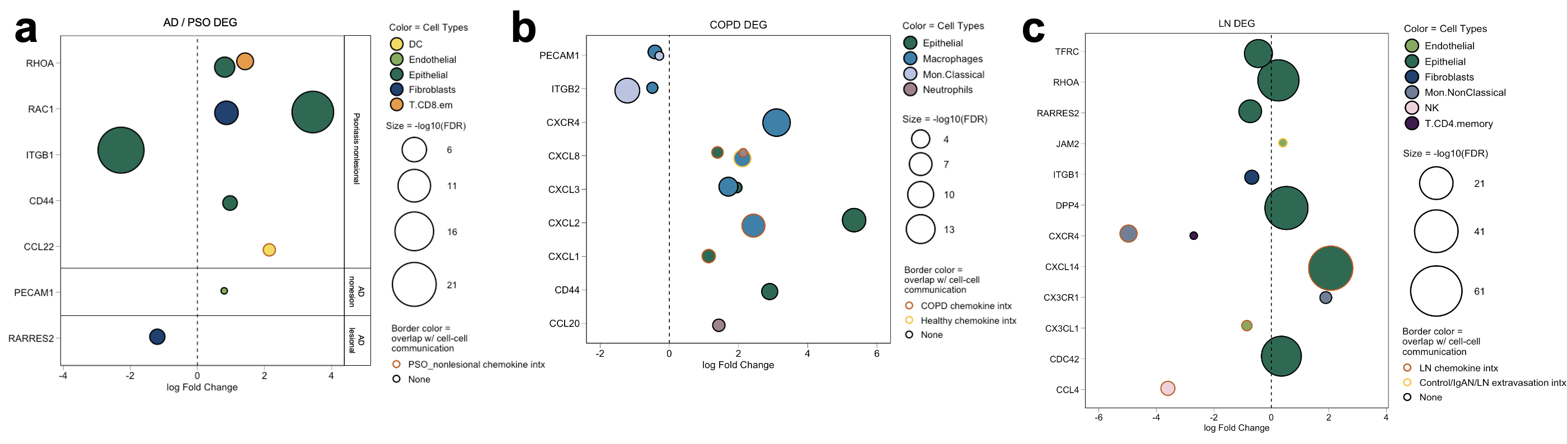

Supplement: Supplementary file 2 [file Image1.jpg]
